# Physical activity behaviors and attitudes among women with an eating disorder: a qualitative study

**DOI:** 10.1186/s40337-021-00377-w

**Published:** 2021-02-10

**Authors:** Jennifer Brunet, Francis Del Duchetto, Amanda Wurz

**Affiliations:** 1 School of Human Kinetics, University of Ottawa, 125 University Private, Montpetit Hall, Room 339, Ottawa, Ontario K1N 6N5 Canada; 2 Cancer Therapeutics Program, Ottawa Hospital Research Institute, 1053 Carling Avenue, Ottawa, Ontario K1Y 4E9 Canada; 3 Institut du savoir Montfort, Hôpital Montfort, 713 Montreal Road, Ottawa, Ontario K1K 0T2 Canada; 4 Present address: Université du Québec à Trois-Rivières, 3351 Boulevard des Forges, Trois-Rivières, Québec, G8Z 4M3 Canada; 5 Faculty of Kinesiology, University of Calgary, 2500 University Drive, Calgary, Alberta T2N 1N4 Canada

**Keywords:** Exercise, Women, Anorexia nervosa, Binge-eating disorder, Bulimia nervosa, Thematic analysis

## Abstract

**Background:**

Physical activity (PA) is an integral part of healthcare for the general population and individuals with psychiatric disorders. However, PA is significantly more complex for and related to both healthy (e.g., reduced anxiety and depressive symptoms) and unhealthy outcomes (e.g., intrusive, repetitive thoughts that lead to abnormally high levels of PA) among women with an eating disorder (ED). Consequently, many healthcare professionals recommend abstinence from PA during ED treatment. Despite this, women may remain engaged in PA during ED treatment or resume PA shortly thereafter. Little has been done to understand women’s PA behaviors and attitudes and to explore how they integrate PA into their lives during and after ED treatment. Thus, this study sought to explore PA behaviors and attitudes among women with an ED.

**Methods:**

Nine women who self-reported receiving an ED diagnosis participated in semi-structured interviews, which were transcribed and analysed thematically.

**Results:**

Six interrelated themes were constructed to represent participants’ PA experiences: *PA as a lifestyle*, *PA can be beneficial*, *How I feel when I can’t do PA*, *Being cautious about PA*, *Reactions to healthcare professionals’ practice of recommending abstinence from PA during ED treatment*, and *PA programming thoughts*.

**Conclusions:**

Overcoming dysfunctional PA behaviors (e.g., engaging in abnormally high levels of PA) and attitudes (e.g., associating PA with caloric expenditure) can be a long and complicated journey for women with an ED. Nevertheless, women with an ED want PA to be integrated into their ED treatment protocol for two key reasons, namely to accrue the benefits associated with PA and to learn how to engage in PA in adaptive and healthy ways.

**Supplementary Information:**

The online version contains supplementary material available at 10.1186/s40337-021-00377-w.

## Plain English Summary

Physical activity (PA) can be beneficial to the physical and mental health of women with an eating disorder (ED). However, little is known about women’s PA behaviors and attitudes. Moreover, many healthcare professionals adopt the practice of recommending abstinence from PA during ED treatment. Despite this, women may remain engaged in PA during ED treatment or resume PA shortly thereafter. This study offers perspectives from nine women with an ED to better understand their PA behaviors and attitudes and to gain insight into how they integrated PA into their lives during and/or after ED treatment. Overall, the women’s accounts reveal that overcoming dysfunctional PA behaviors (e.g., engaging in abnormally high levels of PA) and attitudes (e.g., associating PA with caloric expenditure) can be a long and complicated journey. At the same time, however, they suggest the benefits of PA warrant further consideration. In addition, the women expressed a desire for PA to be integrated into their ED treatment protocol when possible so they accrue benefits and learn how to engage in PA in adaptive and healthy ways.

## Introduction

Eating disorders (EDs) are the most prevalent psychiatric disorder diagnosed in women [1]. The American Psychiatric Association [2] describes eight different feeding and EDs that share defining features, including obsessive preoccupations about food, body shape, and body weight: anorexia nervosa, bulimia nervosa, binge-eating disorder, pica, rumination disorder, avoidant/restrictive food intake disorder, other specified feeding or ED, and unspecified feeding or ED. There are also common behaviors associated with EDs, such as counting calories, frequent weighing/body checking, and hyperactivity [2]. EDs can have a negative impact on physical and psychosocial health. Indeed, physical complications arising from EDs can affect every organ system. Bradycardia, hypotension, anemia, hormonal imbalance, and low bone mass are just some of the negative physical effects commonly observed [3–7]. Also, EDs are associated with high rates of anxiety, depression, body image issues, and obsessive-compulsive disorders [8–10]. Whilst treatments such as pharmacological treatment, nutrition counselling, and cognitive behavioral therapy can help women manage their ED, many continue to experience compromised physical and psychosocial health, consequently re-accessing healthcare for years following their diagnosis [11, 12]. Identifying adjunct therapeutic approaches is necessary to improve both physical and psychosocial health in this population.

The physical and psychosocial health benefits of physical activity (PA), which is defined as any bodily movement that is produced by skeletal muscles that results in energy expenditure^1^ and includes exercise [a subset of PA that is planned, structured and repetitive, with the objective of improving or maintaining physical fitness [13]], suggest it may be a potentially effective therapeutic adjunct to ED treatment to improve health. Literature reviews [14–19] and a meta-analysis [20] conclude that PA can be safe and beneficial for women with an ED, provided their nutritional needs are met. Furthermore, experimental studies report decreased compulsive PA, reduced drive for thinness and bulimic symptoms, improved body satisfaction, facilitated weight gain, enhanced muscular strength, and reversed cardiac abnormalities among women with an ED who engage in PA [21–23]. Additionally, PA can enhance mood, quality of life, and well-being among women with an ED [24, 25]. Last, PA can promote bone health [26], which is beneficial to women with anorexia nervosa because they often have decreased bone mass, impaired bone structure, and reduced bone strength [27].

Though the benefits of PA are notable, women with an ED may struggle to find a balanced approach to PA, with some being highly sedentary [e.g., many with binge eating disorder [28, 29]]. Others, however, engage in abnormally high levels of PA and feel anxious when unable to engage in PA [30, 31]. Some women with anorexia nervosa have described feeling addicted to PA such that their workouts remain unchanged despite social obligations or physical illness/injuries [32, 33]. Women with bulimia nervosa have also reported engaging in PA to compensate for energy surpluses due to binge eating in order to prevent weight gain and/or to regulate negative affect [30, 31, 34]. Anywhere from 30 to 80% of people with an ED engage in abnormally high levels of PA [35, 36], which is problematic because it has been associated with increased hospitalisation length, higher rates of inpatient dropout, compromised treatment efficacy, increased risk of relapse and chronicity, and increased psychological comorbidity [37–41]. Moreover, abnormally high levels of PA are associated with severe physical complications [e.g., absence of menstruation, reduced bone density, chronic energy deficit, reduced levels of growth hormone and insulin-like growth factor, increased levels of the stress hormone cortisol, changes to the microarchitectural skeletal structure, increased fracture risk, increased catecholamines and ghrelin, decreased leptin and thyroid hormones [42, 43]. Because PA is often a significant component of anorexia nervosa and other EDs, continuing to engage in PA before making significant progress during recovery could serve to maintain the obsessive-compulsive aspects of the ED. Consequently, many healthcare professionals may by wary of the risk-benefit ratio associated with PA, hesitate to formally address PA during the course of ED treatment, and understandably recommend abstinence from PA during ED treatment [44, 45]. Nevertheless, the practice of recommending abstinence from PA during ED treatment has been questioned [46] and many women remain engaged in PA during ED treatment or resume PA shortly thereafter to self-regulate, manage problematic feelings, and accrue the benefits associated with PA [33, 47]. Thus, it is important to help women establish a healthy relationship with PA at some point during ED treatment.

Whilst concerns about addressing PA during ED treatment continue to grow given that women with an ED can exhibit dysfunctional PA behaviors and hold different attitudes and motives for PA as compared to women without an ED [48, 49], little has been done to explore in-depth women’s PA behaviors and attitudes and to understand how they integrate PA into their lives during and after ED treatment. An exception is Moola, Gairdner and Amara’s [24] qualitative study in which they interviewed 11 women with anorexia nervosa about their PA experiences. Their findings revealed that women with anorexia nervosa have a complex relationship with PA over the course of their illness, with marked shifts in PA motives – ranging from intrinsically pleasurable pursuits (e.g., walking with friends) to instrumental outcomes (e.g., weight loss). Notwithstanding these contributions, there remains a lack of exploration of women’s perspectives. In an attempt to extend Moola et al.’s [24] research, the aim of this study was to explore PA behaviors and attitudes among women who self-reported having been diagnosed with any type of ED. In turn, the information gained in this study could be used in practice to help women with an ED avoid engaging in abnormally high levels of PA, and instead engage in safe and appropriate amounts of PA once they have been cleared by their healthcare team.

## Methods

A constructivist-interpretative research paradigm, as coined by Denzin and Lincoln [50], was adopted. This paradigm assumes a relativist ontology (i.e., multiple realities exist). Further, it assumes a subjective epistemology (i.e., knowledge is subjective and co-created). As such, a qualitative methodology using dialectical techniques for data collection and interpretation was used herein.

### Participants

Purposeful sampling was used to identify and select information-rich cases who had firsthand experience with an ED. Accordingly, adult women who were willing to communicate their experiences were identified and selected based on the following criteria: (i) self-reported having been diagnosed with an ED by a healthcare professional, (ii) available to participate in this study, and (iii) able to read, understand, provide informed consent, and complete this study in English. Of note, no limit was imposed on type of ED and age to help ensure that the insights and experiences of women with varying ED diagnoses and ages could be captured. Moreover, the rationale behind recruiting women with varying ED diagnoses was to obtain a broad and comprehensive understanding of this phenomenon to lay a foundation for those wishing to continue this line of inquiry.

### Sample size

Experts contend that there is no definitive numerical recommendation for determining sample size in qualitative research. Thus, the widely-used criterion of saturation was used. Specifically, Saunders et al.’s [51] notion of *data saturation* was used such that saturation was monitored continuously throughout recruitment and data collection, and sampling was discontinued when no additional information appeared to be forthcoming (i.e., no new information was heard or uncovered from the ongoing interviews). Following this criterion, potential participants were approached until data saturation was achieved; this was achieved after the seventh interview. However, two additional interviews were conducted with women who had expressed interest in this study before the seventh interview was conducted. Data from all interviewed women were analysed, yielding a total sample size of nine.

### Procedures and data collection

Before recruitment began, ethics approval for this study was obtained from the University of Ottawa and administrative permission was obtained from the Hopewell Eating Disorder Support Centre – a community-based support centre for individuals diagnosed with an ED. A convenience sample of women with any type of ED was recruited by distributing information about this study through staff and placing posters at the Hopewell Eating Disorder Support Centre. Women were given information about this study and screened over the phone by FDD.

After providing written informed consent, participants completed a survey. The survey consisted of three sections. The first section served to collect self-reported personal information including age, ED diagnosis, height and weight (used to compute body mass index; BMI), and stage of treatment. The second section consisted of the Eating Attitudes Test-26 [EAT-26 [52]– a widely used self-report measure of symptoms and concerns characteristic of EDs. The third section consisted of the Exercise Dependence Scale-21 [EDS-21 [53]– a self-report measure widely used to determine risk for exercise dependence based on criteria presented in the Diagnostic and Statistical Manual of Mental Disorders. The data from all three sections were collected to describe the sample.

Once the survey was completed, participants took part in one face-to-face semi-structured interview. All interviews were arranged and conducted by FDD who had personal experience with women with an ED and had received training in qualitative methods (see below). FDD’s knowledge, skills, and experience positioned him ideally to develop rapport with participants and exchange with them about their PA experiences within the context of an ED. During the interview, participants were asked a series of open-ended questions informed by the research aim [54]. After some introductory questions were asked to initiate conversation and establish rapport between FDD and participants, interview questions focused on participants’ PA behaviors and attitudes. Participants were encouraged to deviate from the interview questions and discuss experiences that had significant meaning to them. Follow-up questions and probes were used to elicit more information, ask for further reflection, depth or context, and/or to clarify statements. All interviews ended with an opportunity for participants to make final comments and/or add additional pertinent information. The interviews lasted on average 80 min, were audio-recorded, and took place at the University of Ottawa in a private room. Participants received $25 CAD for participating in this study.

Prior to collecting data for this study, FDD tested the interview guide with three women who had a problematic relationship with food at some point in their lifetime, but fell short of meeting ED diagnostic criteria. The test interviews were conducted to determine if the questions were neutral, clear, facilitated open-ended responses, and flowed well. Additionally, the test interviews provided FDD an opportunity to practice developing follow-up questions and probes. Based on feedback obtained during the test interviews, some of the questions were revised, re-worded, and re-ordered prior to data collection.

### Data analysis

Responses to the survey were analysed using descriptive statistics. For the EAT-26, total scores were computed and participants were classified as *positive* if they scored at or above 20 on the EAT-26, indicating a high level of concern about dieting, body weight, or problematic eating behaviors; otherwise, participants who scored less than 20 were classified as *negative*. For the EDS-21, total scores were computed and participants were classified into one of the three groups using the EDS-21 scoring manual that consists of flowchart decision rules, in which items or combinations of items determined if they should be classified in the dependent, symptomatic, or asymptomatic range on each of the seven Diagnostic and Statistical Manual of Mental Disorders criteria.

The interviews were transcribed verbatim and analysed inductively using thematic analysis [55]. First, JB and AW read each transcript thoroughly several times and took notes of initial thoughts. Next, they generated codes of salient features based on their judgement and relevance to the research aim. Third, they convened to discuss their findings and group similar codes into themes. Fourth, they labelled themes and prepared a table of themes in which subthemes were nested with supporting anonymised quotes to promote resonance. Fifth, they prepared descriptions for each theme.

### Study rigor

Various strategies were followed to enhance the quality of this study [56, 57]. First, the interview guide comprised open-ended questions that allowed participants to enunciate what was important for them. Relatedly, the interview guide was decided upon by two experienced qualitative researchers from different disciplines (i.e., PA psychology and eating disorders) and by FDD allowing for triangulation between researchers. Third, JB and AW engaged in an exhaustive, systematic, and reflective analysis of the data. They also engaged in critical discussions while developing themes and subthemes, continuously reflecting upon and exploring multiple and alternative interpretations of the data. To this end, they listened to each other’s interpretations of the data, offered each other critical feedback, and encouraged reflexivity by challenging each other’s interpretations. Fourth, quotes are presented to give participants a voice, support the analysis, and allow readers to judge the authors’ interpretations of the data. Fifth, participants shared the common experience of being diagnosed with an ED, and were able to communicate their experiences in an articulate, expressive, and reflective manner. Sixth, FDD received in-depth training to develop an understanding of the interview process, to practice his interviewing skills, and enhance his competence when speaking with women with an ED. To this end, he immersed himself in the literature, was given specific training by JB (a knowledgeable and experienced qualitative researcher), completed three test interviews wherein he received critical feedback, and transcribed each interview prior to subsequent interviews to gain insight into how to improve his interviewing skills. Finally, the authors acknowledged and reflected on their preconceptions, life experiences, and knowledge of the literature as they met with participants (FDD), read the transcripts and analysed the data (JB and AW), and interpreted the results (JB, FDD, AW) [see Additional file 1 for reflexivity statement].

## Results

### Participants

Nine women (*M*_age_ *=* 27.9; range = 19–56 years) participated in this study. The survey data (i.e., participants’ age, ED diagnosis, stage of treatment, BMI, EAT-26 scores, and EDS-21 scores) are presented in Table 1. These data demonstrate that participants presented with varying symptoms and concerns that are characteristic of EDs (see Table 1, column 6 for EAT-26 scores), as well as with varying risks for exercise dependence (see Table 1, column 7 for EDS-21 scores).

**Table 1:** Participants’ characteristics (*N* = 9)

Pseudonym	Age (years)	Self-reported ED diagnosis	Stage of treatment	BMI (kg/m^2^)	EAT-26 scores / classification^a^	EDS-21 scores / classification^a^
Sarah	29	Anorexia nervosa	Recovery	19.42	5 / Negative	49 / Nondependent-symptomatic
Teresa	22	Anorexia nervosa	In-treatment	NR	32 / Positive	99 / At-risk for exercise dependence
Diane	56	Binge ED	Recovery	42.28	17 / Negative	40 / Nondependent-asymptomatic
Karen	25	Anorexia nervosa	Recovery	16.82	44 / Positive	58 / Nondependent-symptomatic
Julie	30	Bulimia nervosa	In-treatment	25.88	14 / Positive	44 / Nondependent-symptomatic
Donna	20	Anorexia nervosa	Recovery	19.73	53 / Positive	88 / At-risk for exercise dependence
Anna	30	Bulimia nervosa	Relapse / Seeking treatment	26.57	35 / Positive	60 / Nondependent-symptomatic
Isabelle	19	Other specified feeding or ED	Recovery	24.89	16 / Negative	48 / Nondependent-asymptomatic
Maude	20	Anorexia nervosa	Recovery	NR	7 / Negative	55 / Nondependent-symptomatic

*Notes. BMI* Body mass index, *ED* Eating disorder, *EAT-26* Eating Attitudes Test-26, *EDS-21* Exercise Dependence Scale-21, *NR* not reported

^a^ See **Data analysis** section for classification details

## Main results

The PA behaviors and attitudes collectively held by participants are presented in two sections. The first section, *PA behavior*, describes how active participants were when they were interviewed. The second section, *PA attitudes and experiences*, describes six overarching themes and subthemes (indicated in italics in the text below) that resulted from the analysis of the interviews. This is followed by a third section, *PA programming thoughts*, which describes participants’ thoughts about PA programming for women with an ED. Supporting, anonymised quotes for each section are presented in Tables 2, 3, 4, respectively*.* In the quotes, [...] signifies text that was removed for clarity.

**Table 2:** A snapshot of participants’ PA behavior (*N* = 9)

Very Active	Sarah (anorexia nervosa): “*PA. So it’s my way to get to work. I live about 10 km from where I work, and I usually bike or run to work so it’s my main mode of transportation. And I also started competing again this year in road racing. So I deliberately exercise. Like my training is separate because […] when I’m training, I have something that I need to accomplish like a defined goal at the end of it. And then when I’m commuting, it’s PA to get to work.*” Teresa (anorexia nervosa): “*I basically just do biking and that’s usually like only 25 min. If I’m going to the gym, then I would do like 1 h of full-body weights workout and then if I’m not going to the gym but like going to a class, it tends to be like the group power class that focuses more on strength, but still has a cardio component. I do probably 5 days of PA ranging between 5 days of intentional PA where I’m at the gym and then the other days I would just probably try to get in some more walking. So it ranges from like 1 h to like 1 h and 45 min if I’m at the gym. Also, my goal is to go for a walk every day and the walk is about 40 min and I do 6 to 10 min of interval training every day and when I’m really feeling happy, I also do some Tai Chi and also do some muscle strengthening exercises for certain parts of my body*.” Teresa (anorexia nervosa) Diane (binge ED): “*I go for a walk for about 160 min per week and then interval training is […] 208 min total [per week]. I do a mix of PA and exercise. Exercise simply for exercise is about 50 min per week because it’s the interval training. […] Everything else I call PA but even my walking you know sometimes I walk with people who walk really fast and that’s a cardiovascular workout.*” Julie (bulimia nervosa): “*5 days a week at the moment. I do have a set routine. It’s usually 30 to 45 min. Usually alternating between running or swimming. A lot of cardio. I try to incorporate weight lifting in a circuit training in once a week. And they [activities] are tied to social things so I have a swim class that I joined.*” Donna (anorexia nervosa): “*Now I’m going towards 4 or 5 days a week for I think 1 h and a bit every time. So 5 to 6 h [per week]. […] Usually it’s just going to a gym, going on machines. Rowing or elliptical or treadmill to warm up and then just doing a bunch of weights. It depends what day it is. I do focus on groups. On one day, I just do shoulders, on another day, I do legs.*”
Somewhat active	Karen (anorexia nervosa): *“I walk every day. I do yoga about every day and then again. I’m just up on my feet and active at work and that’s about it*. *Usually 1 h of walking and then 20 or 30 min of yoga*.” Anna (bulimia nervosa): “*1 h 6 days per week. Walking only.*” Isabelle (other specified feeding or ED): “*Now, I guess I just go for a 30-min walk per day or maybe 1 h-long. […] I haven’t been really doing much in the past couple of weeks.*”
Irregularly active	Maude (anorexia nervosa) “*If I want to exercise, I exercise. If I’m too tired or sick, I don’t. [Now, I] do body [workout] and I do cardio.*”

*Note. ED Eating disorder, PA* Physical activity

**Table 3:** *Quotes for themes related to participants’ PA attitudes and experiences*

Themes	Subthemes	***Quotes***
PA as a lifestyle		Sarah (anorexia nervosa): “*It’s probably like an 8 [out of 10]. It’s a big part of everything. I think it’s just a part of a way of being for me. It doesn’t feel like I need to do anything extra. It just feels I need to do something that’s just a part of my normal routine because it’s just been ingrained for so long*.” Donna (anorexia nervosa): “*I think it’s important for your body to move around just so you’re not doing nothing all day. You need to move around. That’s what your body is meant to do. You can’t just sit down and do nothing. I just think it’s important to just workout and be healthy.*” Teresa (anorexia nervosa): “*Exercise is one of the main focuses of my life. I grew up being very active playing sports and stuff. […] I guess [PA is] bringing some identity back to me because I feel like I was always the athletic one in school. It’s important now that I can maybe start to reclaim some of that and be like ‘Hey, you are still sporty and athletic and physically fit or whatever.*” Karen (anorexia nervosa): “*There was just so much sports in my childhood. It was such a big part of my life. Growing up being that active, now I just want to be active because it’s just part of my life, it’s a part of me.*” Diane (binge ED): “*As a kid and young adult I had not made it a priority. Nobody really ever helped me learn how to be physically active as a regular part of my life. But now, I need to make a priority. I’m proud of myself because it’s all coming from me, nobody really ever helped me learn how to be physically active as regular part of my life*.”
PA can be beneficial	Physical benefits	Diane (binge ED): “*I’m much healthier when I’m physically active. My body just feels good. I generally feel even if it’s something that made me tired, I feel more energetic. I mean there are a lot of reasons. It means keeping my body able to move. It means cardiovascular health and it means to have enough strength to do what I want to do. It means flexibility and balance, everything that comes together that allows me to do the things that I want to do*.” Maude (anorexia nervosa): “*I used to have asthma and doing exercise helped me to get rid of it and it keeps me strong and healthy and everything. I also want to live longer. I’m really interested in health in general. PA means taking care my body, my health*.” Anna (bulimia nervosa): “*If I’m being completely honest, I would probably say I prefer exercising to lose weight because it makes me feel better about myself and makes me feel back in control*.”
	Psychosocial benefits	Teresa (anorexia nervosa): “*Like reducing stress or if I’m upset about something then just going for a run is a really nice way to clear my mind. It’s also been a really positive thing in a lot of times in my life. It has kept me, in a sense mentally, not sane* per se*, because I wouldn’t say that I was ever like crazy or anything, but it just really eased a lot of my stress and anxiety around school or my body image*.” Sarah (anorexia nervosa): “*I feel like I have a better time processing emotions if I’m exercising. I just feel calmer overall. I don’t feel anxiety that much, and I don’t feel stressed or worried as much as when I don’t exercise*.” Isabelle (other specified feeding or ED): “*I do think that I can feel a little bit better about myself when I do exercise. When I do exercise, I’m not a total waste of space. I feel like I have a little bit more control over my life. It helps my depression because of the serotonin*.” Karen (anorexia nervosa): “*Like it has a good effect on my brain for sure. It releases endorphins and I feel good doing it. So when I’m active, I feel better. A healthy amount of exercise can be really really good for my mental health. It releases endorphins and I feel good about using my body.*” Diane (binge ED): “*It’s another factor that gives me a great sense of satisfaction and even joy, and as I say pride when I walk*.” Julie (bulimia nervosa): “*I try to make it a social thing so it’s not an isolating activity. I go for a run with a friend, go hiking with groups of friends.*” Maude (anorexia nervosa): “*It has kind of opened up my social circle because I do Zumba in groups.*”
	PA helps with ED management	Teresa (anorexia nervosa): “*Exercise plays such a central role in my ED and my ED kind of consumes my entire life. Exercise has really been like a helpful coping mechanism for me*.” Diane (binge ED): “*My eating is more under control. I feel less necessity to binge and I’m more in touch with the real world. I found that I binge less when I walk in particular. A huge part of the reason that I binge is that I’m feeling empty, lonely, bored, not a very useful person, all kind of negative sort of thoughts. And simply when I get lots of PA, I have far fewer of those thoughts. So it’s a big influence on my kind of eating habits or ED*.” Julie (bulimia nervosa): “*It keeps my appetite regular. I’m less likely to binge and purge. […] It’s a component of the management of my ED. For me, it manages my appetite. I feel like I eat more normal meals at normal times and don’t get as frequent an urge to binge. It manages my moods if I’ve had a rough day at work. Instead of going home and binging, I can go for a run and I feel a lot better afterwards. So it’s been a helpful component of it*.”
How I feel when I can’t do PA	Physical effects	Diane (binge ED): “*If I don’t get enough PA, first of all I get more pain. Secondly, I cannot then do the things I want. All of life becomes a chore if I’m not physically healthy*.” Anna (bulimia nervosa): “*Kind of gross. It’s a lot of like feeling like I’m losing that control I had and there’s not much I can do about it. If it’s for a while, then I can feel myself gaining the weight or losing the muscle. It just kind of feels like it’s downhill from there. It’s not really about not doing PA, it’s more about losing the things that PA gives me. So like putting on a pair of jeans that are getting a little too tight because you’re not doing what you’re supposed to be doing and then kind of just feeling that if you’re laying down on the couch and you’re like ‘I should not be doing this right now. I should be doing something else’ and then it gets pretty stressful*.”
	Psychosocial effects	Donna (anorexia nervosa): “*I feel really bad. If I have 1 day when I don’t workout, I sometimes feel I didn’t do anything even if I went to school or I went to work or something*. […] *I saw some of my friends way less when I stopped going to the gym because that was something social too.”* Sarah (anorexia nervosa): “*I just did not exercise for a week and I noticed a difference in term of like my attitude. It was like more negative and it felt like things were weighting on me heavier. So, it’s difficult to be inactive*.” Karen (anorexia nervosa): “*I’m not happy when I’m stagnant*.” Teresa (anorexia nervosa): “*If I don’t engage in PA and I’m ‘that’ size, then I feel shitty about it and feel like I should be smaller*.”
	Effects on ED management	Teresa (anorexia nervosa): “*I get antsy when I am inactive. 1 or 2 days, then I think ‘I am busy, I’m doing other things.’ On day 3, I think ‘I’m not taking care really. I should probably be getting back onto my routine.’ By day 4, I think ‘I’m having issues managing my ED.’ I feel if I am not exercising, it is not a compulsion, but I can recognise that I have trouble managing my mood, trouble recognising my hunger cues. All these things that come up when I am not exercising regularly*.” Sarah (anorexia nervosa): “*I know that the times where I know I’m going to be inactive, are times where I have to be vigilant of relapse*.”
Being cautious about PA	Changing PA habits	Julie (bulimia nervosa): “*Although exercise helps, I don’t want it to become something that I focus on to a point that it’s detrimental. So, I am more conscious of the fact that if I am exercising for more than 30 or 45 min, it is being excessive. Also, I am conscious of trying to go to the gym 3 times a week*.” Sarah (anorexia nervosa): “*I think it’s just being really careful and honest with myself about where I’m at because sometimes I want to say like ‘10 more minutes but then I have to reel myself back in.*” Teresa (anorexia nervosa): “*Now, it’s definitely not as intense as it used to be. […] I take rest days every week and I limit the amount of time I spend at the gym, I limit the intensity of my exercise. I don’t force myself to go when I really don’t want to go*”. Maude (anorexia nervosa): “*I like doing it so I’ll try to make time for it, but if I really don’t have time or I’m tired or whatever, I’m like ‘Okay, no you have to listen to your body’ and I don’t do it. My body knows best*.” Donna (anorexia nervosa): “*I make sure that I don’t go 2 days in a row. So, it’s either every second day or you know. I still don’t spend as much time as I used to go. I do different things. I’m not just on the cardio machines for 60 min at the highest.*” Karen (anorexia nervosa): “*I loved running before the onset of my ED and at certain points in my recovery, but right now because I’ve only been out of the hospital for 3 months, I don’t feel that I can run yet because I feel that if I run it will turn into a symptom. But I hope that one day I can run again and not feel it’s for my ED and that I actually enjoy it*.” Diane (binge ED): “*I’m not someone that goes overboard on PA. I don’t really push myself. I’m not a marathon runner. I wouldn’t go hiking for 8 h. I don’t climb mountains. I don’t do anything extreme*.” Anna (bulimia nervosa): “*I don’t feel that I’m overactive now because they say to get 1 h of activity per day. That’s what I’ve heard. And I don’t feel like I’m overdoing myself, like overworking myself. It doesn’t feel excessive to me*.”
	Re-thinking what PA means	Sarah (anorexia nervosa): “*I think when I was at the height of my ED, all I thought about was running or doing whatever burns the most amount of calories possible. [Now] I hit my steady weight. I’m aware of how many calories I’m burning while I’m running, but it’s more to figure out what else I need to eat throughout the day*.” Teresa (anorexia nervosa): “*There’s still that mindset that I’m exercising to burn calories or I’m exercising to manipulate my body. […] but [I try to find] things that are fun to do to take the focus off of burning calories*.” Donna (anorexia nervosa): “*I still find I always switch to cardio machines so I can see how many calories I’m burning. For some reason, I have to know how many calories I’m burning. […] And when I walk, I think of the calories I am burning, but I don’t think of the actual number. I’m just thinking ‘I’m burning calories’.*” Karen (anorexia nervosa): “*I can very quickly think about it in terms of how much calories I am burning*.”
Reactions to healthcare professionals’ practice of recommending abstinence from PA during ED treatment		Julie (bulimia nervosa): “*Because of my ED, it [PA] has actually been recommended to me by my therapist as a means of managing my mood, anxiety.”* Teresa (anorexia nervosa): “*I get where they are coming from. I understand that when you exercise, you are burning calories and when you want to gain weight because you are underweight, you probably shouldn’t keep exercise because you’re just going to prolong the process of gaining weight*.” Anna (bulimia nervosa): “*It felt horrible. I was so upset. I was really, really upset and then actually a couple of days later, I was thankful. I was so thankful that she did that because I couldn’t stop myself from doing it [PA]*.” Maude (anorexia nervosa): “*It makes sense, but you’re pissed when you’re in that state and you’re told not to do exercise. But in the long run, it’s like you had such a pattern and a mindset around exercise that you kind of need to break to reframe and get back to it in a different mindset. So I don’t 100% agree with it, but I’m not against it at all*.” Karen (anorexia nervosa): “*I don’t fully agree with the zero activity treatment. It’s been proven over and over again that PA is good for people. So I don’t understand why for people with EDs all of the sudden, it’s not a good thing. I really struggled without it and I found it really annoying because I think it’s going far in the other direction. I don’t think the way of treating that is to eliminate exercise completely because the second you get out the hospital, you want to do it obsessively instead of reintroducing it in your life as something that can be a healthy part of it*.” Donna (anorexia nervosa): “*It sucked that I couldn’t be there and do those things that normal people do, that my friends do and everyone around me is doing.*” Isabelle (other specified feeding or ED): “*I don’t think I would suggest ‘no PA at all’. I would suggest like 15 min walks or you know like half an hour of yoga or something like that. Something where you’re not putting too much pressure on your body, but at the same time you still can do healthy things for you.*”

*Notes. ED* Eating disorder, *PA* Physical activity

**Table 4:** Quotes for theme related to PA programming thoughts

Theme	***Quotes***
PA programming thoughts	Julie (bulimia nervosa): “*Individuality, not necessarily in an ED but just in terms of people’s preferences for the PA they do. People will always have things that they prefer*.” Isabelle (other specified feeding or ED): “*I think that, in general, things that are personalised for people are always more helpful because it’s based on you instead of based on the general demographic.*” Sarah (anorexia nervosa): “*I would try to keep it looser. I guess it would be pretty similar to what I’m doing now just because that’s where my goals are and that’s where I’m able to maintain it.*” Maude (anorexia nervosa): “*Everyone who has an ED is just so so different. So, what might be particularly helpful for someone is probably not going to be helpful for another person*.” Teresa (anorexia nervosa): “*Everybody’s intentions are different and you can’t group all people with an ED into one basket and say ‘Hey, this is your exercise program’ because one person has an exercise compulsion and one hasn’t, and one person is going to hate exercise no matter what and somebody is going to like it. So, it’s got to be different*. *I think that there’s going to be more commonalities between the ED subtypes, but I do still think an individual program is a lot more conducive to healing. […] I think a big part of it is trying to change the mindset from doing PA because you feel like you have to to because you want to and you enjoy it so finding things that are fun to do. […] More leisurely things focused more on actually enjoying it and having fun and finding pleasure out of it.*” Teresa (anorexia nervosa): “*I would hate that. I would never want to be in an environment where I’m exercising with a bunch of other people who have EDs, especially in a class like setting. It would be quite the train wreck.*” Sarah (anorexia nervosa): “*Every time you have a bunch of people with an ED in a room together it’s already a little bit of tension. […] There’s competitiveness there because of the competitiveness and the comparisons*.” Donna (anorexia nervosa): “*It would be pretty triggering to see people who are smaller than me or workout more than me. Just because you’re always so competitive and you want to be the smallest one.*” Julie (bulimia nervosa): “*I do like an environment where it’s not a very competitive thing or where you’re not comparing yourself against others. For me, that’s something that’s important.*” Diane (binge ED): “*I would want it to be a group that is very inclusive that accepts that we all have different abilities and that were all different on a given day and all different from each other. If I was going to do it with other people, I would really want it to be inclusive and accepting essentially.*”

*Notes. ED* Eating disorder, *PA* Physical activity

### Section 1: PA behavior

Participants were generally committed to PA regardless of their ED diagnosis and stage of treatment, though their behavior was highly variable. Participants were categorised inductively based on JB and AW’s interpretations of their responses to questions asked during the interview into one of three overlapping categories (see Table 2). Sarah, Teresa, Diane, Julie, and Donna were categorised as *very active* given the extent of their involvement in PA, which included casual walking as well as more intense and targeted PA. They were consistently participating in high levels of structured PA throughout their week, and the activities they engaged in were mostly done with the intent of improving their cardiovascular endurance, muscular endurance, muscular strength, and flexibility. Karen, Anna, and Isabella were categorised as *somewhat active* as they described themselves as participating in weekly PA that usually consisted of walking (or other light-intensity PA). Though these three women sometimes engaged in PA to improve their cardiovascular endurance, muscular endurance, muscular strength, and flexibility, they mostly engaged in PA with the intent of staying mobile and feeling good. Finally, Maude was categorised as *irregularly active*. She commented that PA was not prioritised over other things in her life and engaged in minimal amounts of PA without a purpose.

### Section 2: PA attitudes and experiences

#### PA as a lifestyle

This theme captures the enduring and important role of PA within participants’ lives. Participants saw being active as essential to taking care of themselves. The positioning of PA as an important and central aspect of their lives was connected to their childhood experiences with PA and/or their ED. Participants described how their experiences with PA during their younger years and at the height of their ED impacted their attitudes toward PA now. However, for Diane, the importance attached to PA and the notion of PA as a lifestyle was new.

#### PA can be beneficial

Participants described how PA positively impacted their physical and psychosocial health, and explained that PA helped them manage their ED. In terms of the *physical benefits*, the favorable impact PA had on participants’ health was clear, regardless of their ED diagnosis. In addition to maintaining their general health, participants described how PA helped them self-manage pre-existing medical conditions, and ultimately extend the length of their life. Additionally, they were acutely aware of how engaging in PA made them feel, and attributed periods of “feeling good” to their participation in PA. Weight loss, looking more toned, having better muscle definition, and improving appearance were also viewed as benefits of engaging in PA, which in turn allowed them to feel better.

In terms of the *psychosocial benefits*, participants described a range of psychological benefits associated with engaging in PA, including reduced stress, depression, and anxiety, adaptive emotional regulation, enhanced body image, as well as feeling a greater sense of control, accomplishment, self-acceptance, competence, confidence, and self-esteem. Participants offered that PA helped them manage stress and anxiety by providing them an opportunity to clear their minds. Some described that PA helped them feel better about themselves because it gave them a sense of control and helped them manage their depression. Moreover, participants discussed feeling accomplished and proud of themselves when they engaged in PA, and said they felt “much healthier and happier” when they engaged in PA. Social benefits of engaging in PA were also described. Although some participants enjoyed engaging in PA to have time to themselves, others saw engaging in PA as an opportunity to avoid isolation and connect with family and friends. For these participants, PA was seen as a way to forge new friendships, and they enjoyed their broader social network as a result of participating in group-based PA classes.

Participants were of the perspective that *PA helps with ED management*. The specific benefits reported, which differed from the aforementioned general benefits, related to coping with ED triggers (e.g., negative emotional states referring to feelings of anxiety, low mood, depression). For instance, participants explained that PA helped them manage some of the negative thoughts that contributed to their ED. Some said that PA was beneficial to them in managing their mood and hunger cues. As well, when discussing their thoughts about the role of PA in their ED, they explained that it helped them control and manage specific ED behaviors (e.g., binging, purging).

#### How I feel when I can’t do PA

No matter their ED diagnosis, participants felt that being inactive negatively affected their physical and psychological health, and hindered their ability to manage their ED. In terms of the *physical effects*, they described how being inactive adversely impacted their body weight. In terms of the *psychosocial effects*, they explained that being inactive took a big toll on them, negatively impacting their general well-being. Indeed, they clearly articulated that their mood and body image were tied to their PA behavior. Participants also explained that being inactive made them feel agitated and antsy, which adversely affected their *ability to manage their ED.* Additionally, they feared relapsing during periods of inactivity and did not see their friends as much.

#### Being cautious about PA

Most participants discussed *changing PA habits,* which stemmed largely from their concern about being “overactive.” For them, PA had dominated their life at the height of their ED. Now, they described seeking, or in some cases having found, a more balanced approach to PA. Overall, they felt their PA behavior was no longer dysfunctional; rather, it was now at a level they were content with. However, this only came as a result of hard work, self-monitoring, and self-imposed boundaries. Participants boundaries involved setting limits on the time spent at the gym, scaling back on intensity, avoiding certain types of PA, varying types of PA, and skipping PA at times of need (e.g., when they were tired or sick). Though most participants exerted a degree of vigilance over their PA, some did not feel this was necessary because PA was not associated with their ED and they did not obsess about PA or engage in abnormally high levels of PA.

For participants for whom PA had dominated their life at the height of their ED, insights were offered into how they were *re-thinking what PA means.* They explained that their proneness to obsess about PA and engage in abnormally high levels of PA in the past was influenced by a dominant discourse of PA in the media – PA burns calories. As a result, they were cognisant of calories burnt by PA and saw caloric expenditure as a key reason for taking part in PA in the past, especially at the height of their ED. Yet, some had managed to distance themselves from this thinking, and others who were still hung up on the idea that PA burns calories were actively trying to move away from that thinking. They noted that the shift in thinking was facilitated by instead taking part in fun, enjoyable low-intensity PA, and using PA to connect with nature and other people.

#### Reactions to healthcare professionals’ practice of recommending abstinence from PA during ED treatment

Participants recounted that their healthcare provider recommended they abstain from PA during treatment, with the exception of Julie who was diagnosed with bulimia nervosa and was encouraged to engage in PA to help her manage her mood*.* Participants generally understood the rationale behind the practice of recommending PA abstinence during ED treatment. Some even appreciated being told to abstain from PA at the height of their ED once their initial reaction of being upset dissipated. This said, Karen and Donna maintained stronger, opposing feelings against the practice of recommending PA abstinence.

Regardless of the feelings participant had, they commented that having to abstain from PA during ED treatment and not receiving support to address their dysfunctional PA behaviors and attitudes from their healthcare team made it difficult for them to initiate or maintain healthy PA patterns. Participants explained how support to manage and integrate PA into their lives would be useful during ED treatment. They also felt that being encouraged to engage in less intensive and shorter bouts of PA when it would be safe would be more helpful to learn how to safely engage in PA than receiving recommendation to abstain from PA altogether.

### Section 3: PA programming thoughts

When asked to describe their thoughts about PA programming for women with an ED, participants highlighted the necessity of customised, individually tailored PA programs based on personal preferences, abilities, and goals. Beyond this, participants stressed the importance of tailoring programs based on each women’s ED symptoms and that PA programs should be focused on showing women with an ED that PA can be a fun, relaxing, mood-modulating, and overall healthy activity.

Though participants recognised that PA can provide social opportunities and facilitate engagement with family and friends, they expressed hesitation and worry about participating in PA with other women with an ED. Participants explained that they would dislike an ED-specific group-based PA program, explaining that being around other women with an ED would likely heighten their natural desire to compare themselves to others, and thus foster a judgmental, competitive environment. Rather, they proposed that if PA programs are to be offered to women with an ED in a group setting, care would be needed to stop women from comparing themselves to others and learn to appreciate others.

## Discussion

The aim of this qualitative study was to explore PA behaviors and attitudes among women with any type of ED. The results revealed that there are important risks associated with PA (e.g., engaging in abnormally high levels), and that overcoming dysfunctional PA behaviors and attitudes can be a long and complicated journey for women with an ED. At the same time, the general assertion that PA confers physical and psychosocial benefits was confirmed. As well, the results suggest that PA may help women with an ED cope with ED triggers (e.g., negative emotional states), manage ED behaviors (e.g., binging, purging), and reduce the risk of relapse. Accordingly, healthcare professionals should remain open to women’s desire for PA support during ED treatment and help them learn to engage in PA in adaptive and healthy ways so as to allow them to reap benefits and better manage their ED. In doing so, it will be critical to address the dysfunctional PA behaviors and attitudes women with an ED hold that can lead them to engage in abnormally high levels of PA, and to investigate the specific benefits of PA for women with different ED diagnoses. Moreover, given that some issues raised with respect to PA behaviors and attitudes during the interviews may be linked with specific EDs and/or stage of treatment, potential variations in women’s experiences via subgroup analyses according to ED diagnosis and/or stage of treatment warrants further investigation.

Participants admitted to engaging in abnormally high levels of PA in the past. They used terms such as “compulsive exercise,”^2^ “unhealthy exercise,” “over-exercise,” “overactivity,” and “exercise dependence” when describing their PA behavior at the onset and/or height of their ED. This aligns with reports that suggest the prevalence of dysfunctional PA behavior varies between 30 and 80% [35, 36], and that it is higher among adults with anorexia nervosa than in healthy controls [see [58] for a review]. Collectively, these findings highlight the need to address women’s dysfunctional PA behaviors and attitudes to help them establish healthy PA levels and cope effectively with periods of inactivity. There is promising evidence that adding structured PA consisting of varied, low-intensity activities to treatment, offering cognitive behavior therapy, and/or providing physical therapy that follows guidelines [14] may reduce dysfunctional PA behaviors and attitudes in women with an ED [59–61].

Although participants were only interviewed once, they described that their PA behaviors and attitudes changed over time and became less dysfunctional. They explained that they participated in various, less intensive activities, and engaged in PA less often and for shorter bouts. Their accounts show that changing PA behaviors and attitudes was a complex and taxing endeavor, which in some cases was still ongoing, but that adopting new motives for PA (e.g., to socialize, regulate emotions, enjoyment, improve health and quality of life, manage ED triggers and behaviors) and avoiding activities that trigger dysfunctional PA was helpful. The finding that participants’ attitudes, not just their behaviors, changed is important given the recently reported paradox observed in people with bulimia nervosa and binge eating disorder [62], which demonstrates that people may score high on compulsive PA instruments alongside low levels of PA because they maintain obsessive beliefs of PA. In contrast, women in this study seemed to lower both their levels and their obsessive beliefs of PA. As this coincided with them adopting PA motives that align with intrinsic origins, encouraging women with an ED to engage in PA for intrinsic reasons (i.e., fun, enjoyment) may help to address dysfunctional PA attitudes, as well as actual PA behavior.

Whilst society in general is bombarded with messages that PA is health-enhancing, such messaging can mask the ways in which PA may contribute to the development and/or maintenance of an ED, and that in some cases PA can be associated with adverse consequences [e.g., lowered BMI, maintained obsessive-compulsive aspect of ED [30, 63]. Anywhere from 30 to 80% of people with an ED engage in abnormally high levels of PA [35, 36], which can have dangerous and possibly life-threatening consequences (e.g., physical damage to the heart and muscles, electrolytic imbalances, de-myelination of the nerves, increased risk of fracture, fatal heart fibrillation). For these reasons, healthcare professionals understandably may adopt the practice of recommending abstinence from PA during ED treatment, especially if they see signs of dysfunctional PA. Participants in this study generally understood that abstinence from PA at certain stages of their treatment was prudent (especially in light of ED-induced medical complications), though a minority maintained that the practice of recommending abstinence from PA during ED treatment ignores the notion that PA is good for health. This likely harks back to the pervasive messaging that people need to engage in PA to be healthy. Indeed, alongside their expression of discontent, they argued that PA is a healthy pursuit and that refraining from PA during ED treatment would equate to becoming unhealthy. As such, it may be important to clarify the rationale for PA abstinence and to help women discover new adaptive coping mechanisms to manage their physical and psychosocial health – underscoring that living a healthy and long life can be realised through many avenues – not just PA.

On the other hand, it is imperative for women to develop a healthy relationship with PA at some point in their life, and thus it is important to formally address PA during the course of ED treatment. Results from this study suggest that recommending abstinence from PA was viewed as failing to provide women with the help they need to engage in PA safely and avoid engaging in abnormally high levels of PA. So while abstinence from PA may be necessary at certain stages of treatment (especially during weight restoration), closely monitored, nutritionally supported therapeutic PA can be safely introduced and confer numerous benefits for women during or after ED treatment [20]. This said, more research is needed to understand how best to help women break the rigid attitudes they may have towards PA, and help them truly enjoy PA as a fun, relaxing, mood-modulating, and overall healthy activity.

### Practical implications

Some women in this study admitted to engaging in abnormally high levels of PA in the past, and two seemed to ignore the potential adverse effects of continuing to engage in PA and/or did not consider their PA behavior a problem warranting treatment; rather, they maintained the belief that PA is always good. Thus, healthcare professionals should remain attuned to dysfunctional PA behaviors and attitudes, and formally address these during ED treatment. Whilst there is little evidence on how to address dysfunctional PA behaviors and attitudes, cognitive behavioral therapy and behavioral strategies (e.g., contingency management, rewarding PA abstinence or low levels of PA) have been recommended [64]. As well, strategies to promote intuitive movement (i.e., movement performed with attention, purpose, self-compassion, acceptance, awareness, and joy, and that focuses on becoming more connected, healthier, and stronger) are emerging as promising, though they require further investigation.

Related to the previous point, careful attention should be paid to women who use PA to control and manage their ED symptoms and behaviors. Although participants in this study shared that engaging in PA helped them regulate their ED symptoms and behaviors, there is the risk that they could fall back into their previous dysfunctional PA behaviors and engage in abnormally high levels of PA but justify it as ‘harmless’ in comparison to other ED behaviors (e.g., binging, purging) or perhaps brush it aside as ‘mild’ compared to their previous PA behavior. Accordingly, ensuring women participate in PA for non-ED related reasons (e.g., to socialize, regulate emotions, improve health and quality of life) and addressing the underlying issues associated with women’s ED will be necessary to treat dysfunctional PA behaviors and attitudes, and promote optimal healing, recovery, and relapse prevention. To this end, it might be valuable to offer post-PA debriefing sessions where women can discuss their experiences during PA and confirm that sessions were fun, enjoyable, relaxing, mood-modulating, and overall deemed healthy.

In addition, even if the practice of recommending abstinence from PA is understandable given the risks, the results from this study suggest that the benefits of PA may warrant its integration during ED treatment, contingent upon treatment compliance and when weight requirements and/or weight gain goals are met. When integrating PA, individually tailored approaches that include a graded PA program beginning with short bouts of low-intensity PA are suggested [65]. Furthermore, bearing in mind that women with an ED are sensitive to what others around them are doing and seem motivated to do more than others, it is not advised to have them engage in PA with others with whom they can compare themselves with.

Moreover, when inquiring about women’s PA history, understanding their preferences and abilities is important to facilitate the development of programs that favor enjoyment and that could potentially yield more positive experiences. Beyond this, ensuring PA programs and opportunities educate women and equip them to self-manage their PA behavior, while mitigating competition, may also encourage healthy PA integration. Accordingly, those offering PA opportunities to women with an ED should remove means of tracking progress (e.g., equipment that provide caloric expenditure data), incorporate activities that necessitate teamwork, and seek to foster an inclusive environment. Finally, there is a need to train PA professionals using guidance such as the Safe Exercise at Every Stage Guidelines. These guidelines can help PA professionals make informed decisions on how to help women safely (re)engage in PA during ED treatment and may even be used by healthcare professionals when having discussions about PA with their patients.

### Study limitations

The limitations of this study should be kept in mind when interpreting the findings. First, participants may have been reluctant to disclose certain experiences because they were interviewed by a male. Second, participants self-reported their PA behavior, ED diagnosis, and stage of treatment, and no contact was made with healthcare providers to confirm ED diagnoses. Consequently, there may be errors due to the self-reported nature of these data. Indeed, methods of self-report have been criticised with ED patients because they are known to hide/underestimate their symptoms [66]. Third, the depth of the information gathered on PA behavior varied in richness across participants, which made it difficult to categorise some participants based on both quantitative (i.e., intensity, frequency, duration, and type of PA) and qualitative (i.e., motives for PA) elements. Relatedly, participants were categorised into one of three categories based on JB and AW’s interpretations of their responses to some of the questions asked during the interview. To eliminate the possibility of misclassification and to allow for more precise classification in future studies, additional questions that require participants to report full PA behavior details should be asked. Fourth, this study was conducted in a Western, economically developed country with women who self-reported being employed and had a postsecondary education. Fifth, most participants were between 19 and 30 years of age, making it necessary to further explore women’s experiences across the lifespan as additional perspectives might have emerged if more middle-aged and older women had participated. Accordingly, recruitment strategies may need to be tailored to different age groups to ensure middle-age and older women with an ED are recruited into PA studies as they have the potential to generate data that can be translated into more effective ED management. To this end, exploring strategies to recruit middle-age and older women with an ED into PA studies is crucial to advancing this area of research. Sixth, though questions asked about change over time, participants were only interviewed once. Last, although data saturation was achieved, it is possible that continuing to interview additional women may have further contributed to the understanding of one or more themes.

## Conclusions

Based on the current study, as well as previous literature [14–19], PA is associated with numerous benefits for women with an ED. However, PA behaviors and attitudes are highly complex for this population. As such, findings suggest that treating women with any type ED and supporting them in their recovery will require holistic approaches that include education alongside opportunities to discover varied, intrinsically motivated low-intensity PA options. This said, it is clear that more work is needed to understand how healthcare and PA professionals can work together to empower and support women with an ED and to delineate if and how these findings differ amongst samples comprised of women with specific EDs, who are at specific stages of treatment and recovery, and who are younger or older. A next logical step would be to explore experiences among subsets of the ED population and to engage stakeholders (e.g., women with an ED, healthcare professionals, PA professionals) to review the current findings and co-develop programs to offer women with an ED support for PA.

### What is already known on this subject?

Among women with an eating disorder (ED), physical activity (PA) is related to both healthy and unhealthy outcomes. Consequently, PA is typically restricted during treatment. Yet, women may remain engaged in PA during treatment or resume PA shortly thereafter. There is a critical need to understand women’s PA behaviors and attitudes and to explore how they integrate PA into their lives during and after ED treatment.

### What your study adds?

The exploration of PA behaviors and attitudes among women with an ED revealed that overcoming dysfunctional PA behaviors (e.g., engaging in abnormally high levels) and attitudes (e.g., associating PA with caloric expenditure) can be a long and complicated journey, but that the benefits of PA warrant further consideration. It also revealed that helping women engage in PA in adaptive and healthy ways when possible during ED treatment may be beneficial.

## Supplementary Information

**Additional file 1. **Reflexivity statement.

## Data Availability

The data cannot be shared as participants were assured that their data would be kept private and confidential to the extent permitted by law and that only the research team would have access to the data.

## Abbreviations

BMI: Body mass index
EAT-26: Eating Attitudes Test-26
ED: Eating disorder
EDS-21: Exercise Dependence Scale-21
PA: Physical activity

## Footnotes

1 Henceforth, the broader term of ‘PA’ will be used to describe all bodily movement, whether structured or unstructured.

2 The use of the word “exercise” has not been replaced by PA when quoting participants.
