# Supplementary material for: Physical activity behaviors and attitudes among women with an eating disorder: a qualitative study
Source: J Eat Disord. 2021 Feb 10;9:20. doi: 10.1186/s40337-021-00377-w (PMC7877068; doi:10.1186/s40337-021-00377-w)
Supplement: Supplementary file 1 — Additional file 1. Reflexivity statement. [file 40337_2021_377_MOESM1_ESM.docx]

**Reflexivity**

The researchers’ interpretation of the data is one of the main tenets of a qualitative study. Accordingly, reflexive practices and positioning the researchers within the study are important. The researchers therefore acknowledged and reflected on their preconceptions, life experiences, and knowledge of the literature as they met with participants (FDD), read the transcripts and analyzed the data (JB and AW), and interpreted the results (JB, FDD, AW). As well, the interviewer (FDD), a 21-year old male who was undertaking the work as part of a Bachelor’s degree, prepared a brief summary of his reflections and how they pertain to this study so as to recognize his personal biases that might compromise the interview process. Specifically, he reflected on the personal connection he had to women with an eating disorder (ED). The combination of his experience and his pursuing of a degree in Human Kinetics led him to see the good and the bad of physical activity (PA) for women with an ED. He perceived that PA could be beneficial but could also have serious implications for women’s health and well-being due to the risk of PA dependence. His experiences allowed him to build rapport with participants during the interviews as he was able to empathize with their experiences, as well as probe further on topics he believed might be important to women based on his previous experience. He remained aware of his knowledge throughout, ensuring that his questioning and probing were designed to capture each women’s individual experience.
